# Comparison of the respiratory bacterial microbiome in cats with feline asthma and chronic bronchitis

**DOI:** 10.3389/fvets.2023.1148849

**Published:** 2023-03-27

**Authors:** Melanie Werner, Jasmin Weeger, Lina Hörner-Schmid, Karin Weber, Jelena Palić, Jonathan Shih, Jan S. Suchodolski, Rachel Pilla, Bianka Schulz

**Affiliations:** ^1^Clinic of Small Animal Internal Medicine, Centre for Clinical Veterinary Medicine, Ludwig-Maximilian-University, Munich, Germany; ^2^Clinic for Small Animal Internal Medicine, Vetsuisse Faculty, Zurich, Switzerland; ^3^Vet Med Labor GmbH, Division of IDEXX Laboratories, Kornwestheim, Germany; ^4^Gastrointestinal Laboratory, Department of Small Animal Clinical Sciences, Texas A&M University, College Station, TX, United States

**Keywords:** microbiota, cough, lung, eosinophilic, neutrophilic

## Abstract

**Objectives:**

While feline chronic bronchitis (CB) is known as neutrophilic bronchial inflammation (NI), feline asthma (FA) is defined as an eosinophilic airway inflammation (EI). Feline chronic bronchial disease refers to both syndromes, with similar clinical presentations and applied treatment strategies. Recent studies described alterations of the microbiota composition in cats with FA, but little is known about the comparison of the lung microbiota between different types of feline bronchial disease. The study aimed to describe the bacterial microbiota of the lower respiratory tracts of cats with FA and CB and to identify potential differences.

**Methods:**

Twenty-two client-owned cats with FA (*n* = 15) or CB (*n* = 7) confirmed *via* bronchoalveolar-lavage (BALF)-cytology were included. Next-generation sequencing analysis of 16S rRNA genes was performed on bacterial DNA derived from BALF samples. QIIME was used to compare microbial composition and diversity between groups.

**Results:**

Evenness and alpha-diversity-indices did not significantly differ between cats with FA and CB (Shannon *p* = 0.084, Chao 1 *p* = 0.698, observed ASVs *p* = 0.944). Based on a PERMANOVA analysis, no significant differences were observed in microbial composition between animals of both groups (Bray-Curtis metric, *R*-value 0.086, *p* = 0.785; unweighted UniFrac metric, *R*-value −0.089, *p* = 0.799; weighted Unifrac metric, *R*-value −0.072, *p* = 0.823). Regarding taxonomic composition, significant differences were detected for *Actinobacteria* on the phylum level (*p* = 0.026), *Mycoplasma* spp. (*p* = 0.048), and *Acinetobacteria* (*p* = 0.049) on the genus level between cats with FA and CB, with generally strong interindividual differences seen. There was a significant difference in the duration of clinical signs before diagnosis in animals dominated by *Bacteriodetes* (median 12 months, range 2–58 months) compared to animals dominated by *Proteobacteria* (median 1 month, range 1 day to 18 months; *p* = 0.003).

**Conclusions and relevance:**

Lung microbiota composition is very similar in cat populations with spontaneous FA and CB besides small differences in some bacterial groups. However, with disease progression, the lung microbiome of cats with both diseases appears to shift away from dominantly *Proteobacteria* to a pattern more dominated by *Bacteriodetes*. A substantial proportion of cats tested positive for *Mycoplasma* spp. *via* sequencing, while none of them tested positive using classical PCR.

## 1. Introduction

Feline asthma (FA) is considered a type-I allergic hypersensitivity reaction characterized by eosinophilic inflammation (EI), bronchial hyperreactivity, reversible airway obstruction, increased mucus production, bronchial wall edema, and smooth muscle hypertrophy (1–4). The hallmarks of feline chronic bronchitis (CB) are neutrophilic inflammation (NI) of the airways with local edema formation, hypertrophy of the mucosa and goblet cells, and increased mucus production (3, 5, 6). Both syndromes are often grouped under the umbrella term “feline chronic bronchial disease.” Approximately 1% of the cat population has been reported to be affected by these diseases (7, 8). While the clinical picture of FA has been further studied in recent years, also by establishing allergen-induced models in cats to investigate etiology, diagnostic parameters, and therapeutic options, CB in the cat has hardly been studied and the etiology is still unexplained (9–11). Preceding respiratory infections or changes in the microbial composition of the lung are discussed in humans with CB (12–18). Cats with FA appear to be younger than those with CB, and Siamese cats appear to be overrepresented in some studies, suggesting a genetic predisposition (19–22). There seems to be little difference in clinical presentation between the two conditions (8). Clinical presentation can range from mild clinical signs such as exercise intolerance, tachypnea, and cough to life-threatening dyspnea (20, 21, 23). The classic diagnostic workup of both conditions includes laboratory and radiographic examination, and bacteriological and cytological evaluation of bronchoalveolar-lavage-fluid (BALF) (6, 14, 20, 21, 23–25). In cats with chronic respiratory signs, an increased percentage (>7%) of neutrophils in BALF cytology with physiologic eosinophilic content is suggestive of CB, whereas an increased proportion of eosinophils (>18%) in BALF is considered typical for FA (6, 26). Secondary bacterial infections should be excluded by bacteriological examination in combination with PCR for *Mycoplasma* spp. (27–29). The healthy feline lung has traditionally been considered sterile for a long time. However, the respiratory tract is in constant contact with air from the environment, which inevitably leads to microbial colonization (30, 31). The warm and moist environment of the bronchial tubes and bronchioles provides an environment that allows the establishment of a physiological lung microbiome. The composition of the microbiota in the lower respiratory tract is thought to be determined by the balance of three factors: first, microbial immigration into the respiratory tract; second, elimination of microbes from the respiratory tract; and third, the relative rate of proliferation of each bacterial group, which is determined by regional growth conditions (32). These factors can all be influenced by various processes. However, culture-based detection methods cannot adequately capture the totality of microorganisms found in the respiratory tract (20, 26). Culture-independent methods, such as sequencing techniques, have the advantage of identifying microorganisms that cannot be cultured and are harbored in small proportions (33). The totality of lung microorganisms (including bacteria) evaluated by these methods is defined as the lung microbiome. In human medicine numerous studies have shown that the lung microbiome differs significantly between diseased and healthy individuals (14, 18, 32–36). The lung microbiome may not only influence individual susceptibility for development of respiratory disorders, but may also have an impact on disease progression as well as response to treatment (37). Recently, veterinary studies described alterations of the microbiota composition in cats with spontaneous and induced asthma compared to healthy individuals (31, 38).

Currently, no study is available evaluating the differences in the lung microbiome between cats with FA and CB. The aim of this study was to describe the composition of the bacterial microbiota of the lower respiratory tract of a cat population with FA and CB in more detail and to identify potential differences.

## 2. Materials and methods

### 2.1. Ethical statement

The study was approved by the Ethics Committee of the Faculty of Veterinary Medicine, Ludwig-Maximilian-University (Number: 119-09-04-2018). Cat owners were informed about the study in advance, and written informed consent was signed by each owner before participation in the study.

### 2.2. Study population

This was a prospective observational study, performed between 2018 and 2020. Client-owned cats that were presented to the Clinic of Small Animal Medicine for respiratory signs and received further diagnostic testing including BALF-sampling were eligible for inclusion into the study. Clinical signs considered suggestive for feline chronic bronchial disease included cough, dyspnea, and abnormal breath sounds like wheezing. Cats of any age, sex, and breed were included. Cats were excluded, if they had received glucocorticoids or antibiotics within the last 4 weeks. Chest radiographs were performed in all cats to evaluate lung pattern and to exclude patients with evidence of neoplasia, pneumonia, heart disease, and foreign bodies as causes of respiratory problems. A complete blood count with differentiation and a serum chemistry profile were performed in all cats. Fecal samples from all cats with access to the outdoors were analyzed using the Baermann technique to rule out lung worm infection.

### 2.3. Sample collection

A diagnosis of FA or CB was established by evaluation of BALF cytology. For BALF sample collection, cats were placed under general anesthesia and intubated with a sterile 3.5–4 French endotracheal tube. One to two aliquots of body-warm sterile sodium chloride solution of 3–4 ml (Isotonic saline solution, 0.9%; B Braun Vet Care, Germany) were blindly applied over a sterile polyvinyl catheter (CH 4.5, 1.0 × 1.5; B Braun Vet Care, Germany) that was advanced into the bronchial tree until resistance, then fluid was immediately aspirated back using mechanical suction. BALF was used to prepare slides for cytological examination, and *Mycoplasma*-spp.-PCR and bacteriological examination were performed in all cases. Cats were excluded, if BALF-cytology, *Mycoplasma*-spp.-PCR or bacterial culture indicated bacterial infection or if there was evidence of oropharyngeal contamination with squamous cells or *Simonsiella* spp. bacteria on cytology.

For cytological evaluation, both direct and cytocentrifuged smears were prepared (Hettich ROTOFIX 32A, Tuttlingen, Germany, 5 min at 95 g). The slides were stained with modified Wright's stain after air drying. To assess the type of inflammation, two cytospin preparations and at least two direct smears were assessed. Multiple fields on each slide were evaluated, and differential count was performed on the cytospin preparation that appeared more representative. All slides were evaluated by the same board-certified clinical pathologist, and a differential count of 100 cells was performed on each of several fields. A cutoff value of >20% eosinophils was used to support the diagnosis of FA. Values of >14% neutrophils and < 20% eosinophils were used to establish a diagnosis of CB.

### 2.4. Microbiome analysis

#### 2.4.1. DNA extraction

Three to four milliliter of BAL was further processed within 1. Fifteen microgram acetylcysteine (200 mg/ml) was added as previously described (39), and the liquid was centrifuged at 10,000 g for 10 min. The supernatant was discarded. The pellet was subsequently frozen at −0°C until further use. The pellet was then resuspended in 800 μl lysis buffer (4% Natriumdodecylsulfat, 50 mM EDTA, 500 mM NaCL, 50 mM EDTA, 50 mM Tris-HCl). After incubation at 70°C for 20 min with periodic vortexing, all samples were centrifuged for 5 min at 5,000 g. After adding 200 μl of 10 m Mammoniumacetate to the supernatants, samples were incubated on ice for 5 min, followed by centrifugation at 5,000 g for 15 min at room temperature. One volume of chilled isopropanol was added to the supernatant in a new Eppendorf tube. We incubated the samples at 4°C for 30 min on ice and then centrifuged them at 16,000 g for 15 min. After washing with 70% ethanol, DNA pellets were resuspended in 150 μl of TrisEDTA (10 mM Tris and 1 mM EDTA), followed by 15 μl of proteinase K and 200 μl of ALBuffer (DNeasy Blood and Tissue kits, Qiagen). After the samples had been incubated for 10 min at 70°C, 200 μL of 100% ethanol was added to the tubes. Following the mixing of samples by gentle pipetting, the contents were transferred to a spin column using the DNeasy kit. DNA was purified according to the manufacturer's instructions and eluted in 200 μl of EBbuffer. Using fluorometry (Qubit dsDNA BR assay, Life Technologies, Carlsbad, CA), DNA concentrations were measured, and samples were stored at 80°C and sent on dry ice to Texas A&M University Gastrointestinal Laboratory for sequencing.

#### 2.4.2. Sequencing

The V4 region of the 16S rRNA gene was sequenced at MR DNA laboratory (Shallowater, TX) using primers 515F (5′-GTGYCAGCMGCCGCGGTAA) (40) to 806RB (5′-GGACTACNVGGGTWTCTAAT) (41). Briefly, amplification was performed under the following conditions: 95°C for 5 min, followed by 30 cycles of 95°C for 30 s, 53°C for 40 s and 72°C for 1 min, and a final elongation step at 72°C for 10 min. After amplification, PCR products were checked in 2% agarose gel, and samples were multiplexed using unique dual indices and pooled together in equal proportions based on their molecular weight and DNA concentrations. Pooled samples were purified using calibrated Ampure XP beads and an Illumina DNA library was prepared. Sequencing was performed on a MiSeq following the manufacturer's guidelines. Raw sequences were deposited at the NCBI Sequence Read Archive under project number PRJNA916730.

Using QIIME 2 (Quantitative Insights Into Microbial Ecology 2) v 2019.7 (42), the sequences were processed and analyzed. DADA2 was used to create the amplicon sequence variant (ASV) table after the sequences were demultiplexed (43). Sequences containing < 0.01% of the total reads and assigned as chloroplasts, mitochondria, and low abundance ASVs were removed prior to downstream analysis. Prevalence-based filtering of putative contaminant ASVs was performed by the R package decontam (v0.99.1) (44). A DNA extraction blank was generated contemporaneously and processed in parallel with biological samples as a negative control. ASV tables were used as the input for the Contaminant() function (pss, method = “prevalence,” neg = “is.neg,” threshold = 0.5). Ggplot2 was used to visualize the contaminants generated and contaminants were filtered from the ASV table for further analysis. All samples were then rarefied to even sequencing depth, based on the lowest read depth of samples, to 646 sequences per sample.

QIIME2 was used to evaluate alpha diversity using Chao1 (richness), Shannon diversity, and observed ASVs metrics. Beta diversity was estimated using Bray-Curtis, Jaccard, and unweighted and weighted UniFrac distance matrices, and Principal Coordinate Analysis (PCoA) plots were generated in QIIME2 (45).

### 2.5. Statistical analysis

Statistical analysis was performed using Graph Pad Prism version 9 (Prism v.9, Graphpad Software Inc, La Jolla, California, US). All numerical data were tested for normality using the Shapiro-Wilk-test, and further tests were performed depending on whether they were normally distributed or not. Numerical data are shown as median and range. Age differences between groups were examined with an unpaired *T*-test. Binominal data (sex, occurrence of clinical signs) were evaluated with Fisher's-exact-test. The alpha and beta diversity metrics were computed using Qiime2. Alpha diversity indices (Observed ASVs, Shannon, Chao1) were calculated for each sample.

Comparison of alpha diversity indices (Chao1, Shannon's Diversity, and Observed ASVs) between the two groups FA and CB was performed dependent on normal distribution using unpaired *t*-test or Mann-Whitney-test and compared with each other. To identify compositional differences (beta diversity) in the microbiome between and within study groups, similarity analysis (ANOSIM) was conducted using the PRIMER 7 statistical software package (PRIMER-E Ltd., Lutton, UK), and PERMANOVA was carried out using QIIME2 based on Bray-Curtis metric, Jaccard metric, and the unweighted and weighted UniFrac distance matrices. The Shapiro-Wilk-test was used to assess the normality of individual bacterial abundances. The taxonomic analysis of paired samples was conducted using a 2-way RMANOVA-test. The Benjamin & Hochberg-method was used to adjust *p*-values for multiple comparisons at each taxonomic level. Significance was set at *P* < 0.05.

## 3. Results

### 3.1. Study population

Twenty-two cats were included in the study. There were 15 cats in the FA group and seven cats in the CB group. Median age of all cats was 3.0 years (range 0.5–14.0). Eight cats were female neutered, one female, 13 male neutered. The population included 10 domestic shorthair cats, four Abyssinian, two Siamese, one Ragdoll, one Norwegian forest cat, one British shorthair, one Bengal, one Maine Coon, and one Turkish Van. Table 1 shows the distribution of these parameters between the two groups. Regarding the population parameters, there were no significant differences between the groups. Table 2 shows the primary clinical signs of the cats, the duration of the disease prior to presentation, results of BALF-cytology, and final diagnosis.

**Table 1:** Baseline parameters in cats with chronic bronchial disease.

	**FA (*n* = 15)**	**CB (*n* = 7)**	***p*-value**
Age in years [median (range)]	3.0 (0.5–10.0)	5.0 (1.0–14.0)	0.994
Bodyweight in kg [median (range)]	4.2 (3.0–7.3)	4.0 (2.8–5.0)	0.400
Sex	9 male, 6 female	4 male, 3 female	>0.999
Breed	DSH (8), Abyssinian (1), Bengal (1), British Shorthair (1), Maine Coon (1), Norwegian Forest cat (1), Ragdoll (1), Turkish Van (1)	Abyssinian (3), DSH (2), Siam (2)	

FA, feline asthma; CB, chronic bronchitis.

**Table 2:** Demographic data, clinical signs, BALF-cytology and final diagnosis of cats with chronic bronchial disease.

**Case**	**Breed**	**Age (years)**	**Sex**	**Bodyweight (kg)**	**Environment**	**Cough**	**Dyspnea**	**Wheezing**	**Duration of clinical signs (months)**	**BALF-cytology**	**Diagnosis**
#1	DSH	0.5	f	3.9	Outdoor	Yes	No	No	2	EI	FA
#2	Bengal	0.5	m	4.2	Indoor	No	No	Yes	1	EI	FA
#3	DSH	1	m	4.1	Indoor	Yes	Yes	Yes	4	EI	FA
#4	BSH	1	mc	4.7	Indoor	No	Yes	Yes	3	EI	FA
#5	DSH	2	f	3.2	Indoor	Yes	Yes	No	12	EI	FA
#6	Maine Coon	2	fs	4.1	Indoor	No	No	Yes	0.03	EI	FA
#7	DSH	2	fs	4.4	Indoor	Yes	Yes	No	1	EI	FA
#8	Abyssinian	3	mc	5.2	Indoor	Yes	No	No	24	EI	FA
#9	Ragdoll	3	m	3.0	Indoor	Yes	No	Yes	0.03	EI	FA
#10	DSH	6	f	4.7	Indoor	Yes	No	Yes	48	EI	FA
#11	DSH	6	mc	5.5	Indoor	Yes	No	No	3	EI	FA
#12	DSH	6	mc	5.0	Outdoor	Yes	Yes	No	1	EI	FA
#13	DSH	7	mc	3.7	Indoor	No	No	No	1	EI	FA
#14	Turkish Van	9	mc	7.3	Indoor	Yes	No	Yes	18	EI	FA
#15	Norwegian forest cat	10	fs	3.0	Outdoor	Yes	Yes	No	24	EI	FA
#16	DSH	1	m	4.0	Indoor	No	No	No	3	NI	CB
#17	Abyssinian	2	mc	3.8	Outdoor	Yes	No	No	4	NI	CB
#18	Abyssinian	3	fs	2.8	Outdoor	Yes	No	No	12	NI	CB
#19	DSH	5	fs	4.5	Indoor	Yes	Yes	Yes	5	NI	CB
#20	Siam	12	mc	4.7	Indoor	Yes	Yes	No	60	NI	CB
#21	Siam	13	f	3.2	Indoor	Yes	Yes	No	6	NI	CB
#22	Abyssinian	14	mc	5.0	Outdoor	Yes	No	No	2	NI	CB

f, female; m, male; fs, female spayed; mc, male castrated; DSH, domestic short haired; EI, eosinophilic inflammation; NI, neutrophilic inflammation, FA, feline asthma; CB, chronic bronchitis.

### 3.2. BALF sequence analysis—Alpha and beta diversity

**Figure 1:**
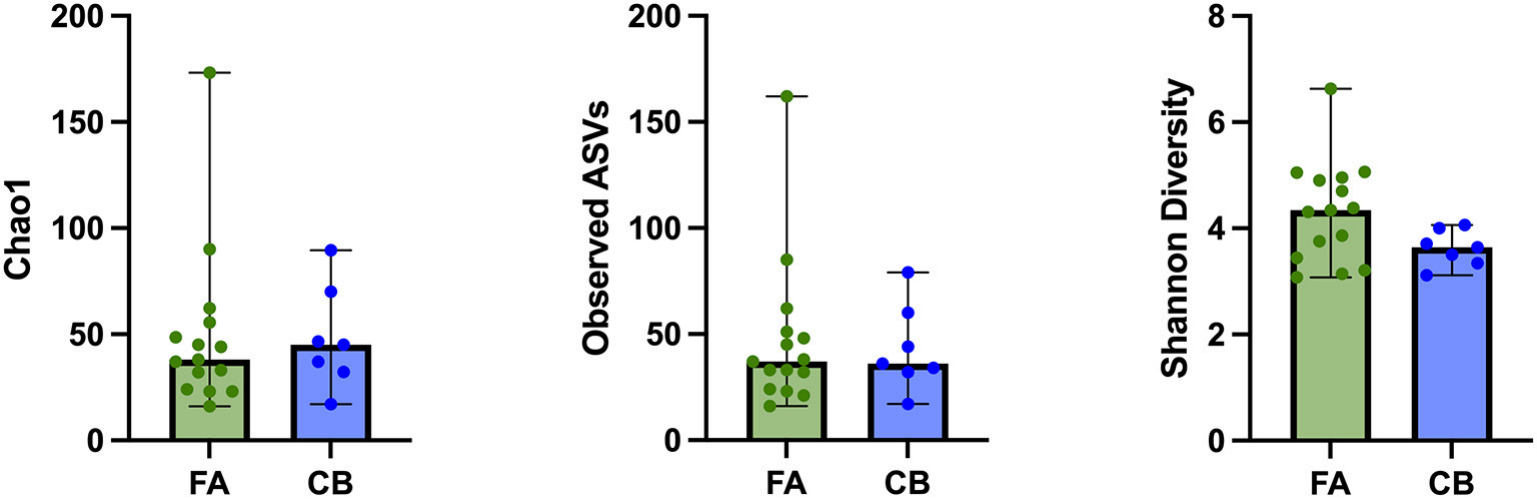
Alpha diversity indices of cats with feline asthma (FA; green dots) and chronic bronchitis (CB; blue dots). Alpha Diversity indices showed no significant difference between cats in the FA and CB group. Median is represented by the upper limit of the colored boxes, range is represented by the upper and lower thin lines.

**Figure 2:**
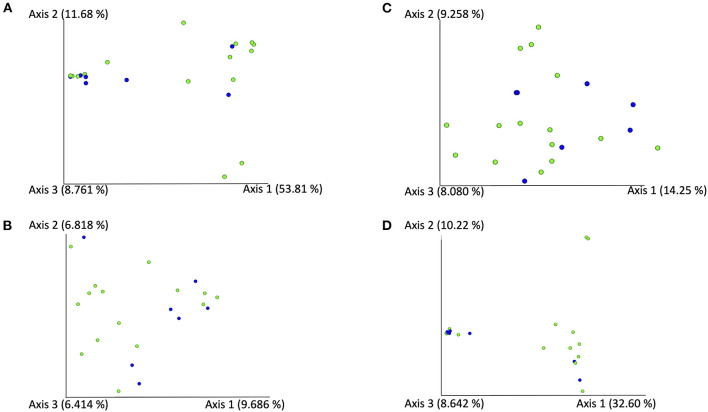
PCoA-Plots [using an unweighted **(A)** and weighted **(B)** Unifrac analysis using the Bray-Curtis **(C)**, and the Jaccard method **(D)**] of BALF-samples of cats with feline asthma (green dots) and chronic bronchitis (blue dots).

In the FA group, the Shannon diversity index median was 4.34 (range: 3.08–6.63), the Chao1 index median was 38 (range: 16.00–173.33), and the observed ASVs median was 37 (range: 16–62). In the CB group, Shannon diversity index median was 3.64 (range: 3.12–4.06), the Chao1 index median was 45.0 (range: 17.0–89.5), and the observed ASVs median was 36 (range: 17–79). Alpha diversity indices and evenness were not significantly different between cats in both groups (Shannon *p* = 0.084, Chao 1 *p* = 0.698, observed ASVs *p* = 0.944; Figure 1). Principal component analysis (PcoA) was utilized to evaluate the beta diversity of the bacterial populations. With the different methods used to determine beta diversity no significant differences in microbiota composition between animals in the FA and CB group on the basis of a PERMANOVA analysis (Bray-Curtis metric, *R*-value −0.086, *p* = 0.785; unweighted UniFrac metric, *R*-value −0.089, *p* = 0.799; weighted Unifrac metric *R*-value −0.072, *p* = 0.823) could be shown. Figure 2 shows the PcoA plots using an unweighted and weighted Unifrac analysis, the Bray-Curtis, and the Jaccard method, respectively. No clustering of cats with CB compared to the FA group can be seen in the graphs.

### 3.3. BALF sequence analysis—Taxonomy results in comparison between FA and CB

**Figure 3:**
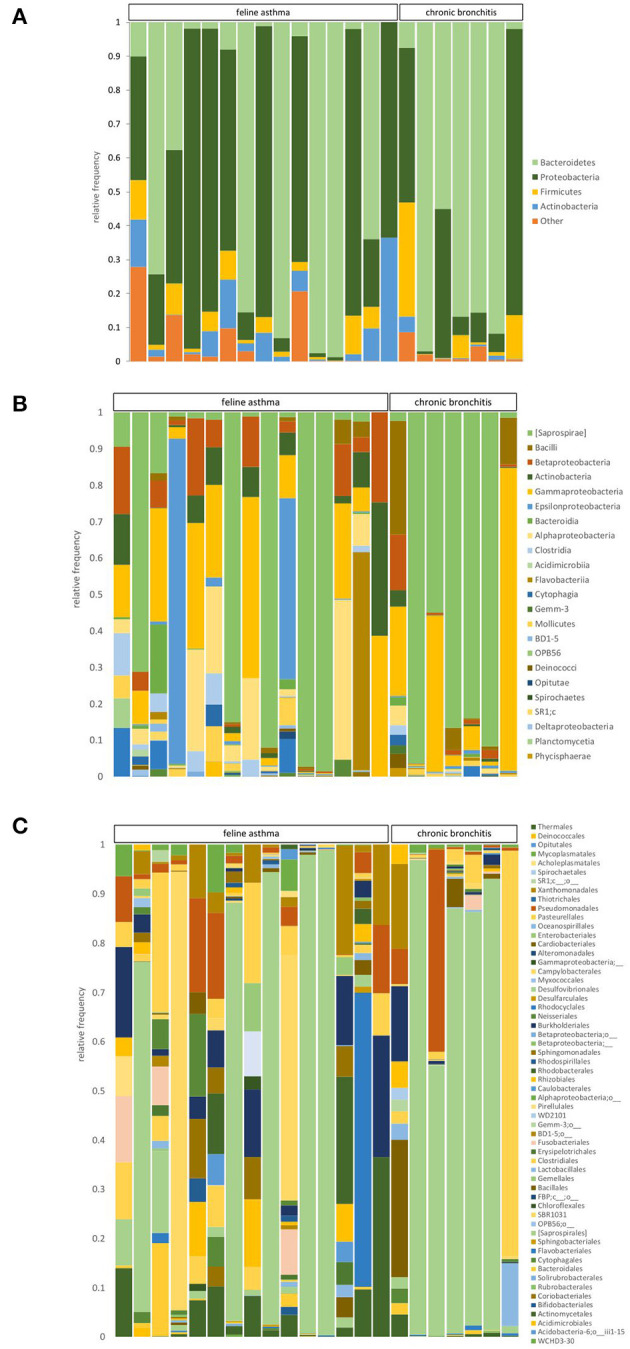
Relative abundance of taxa present in BALF samples of cats with feline asthma and chronic bronchitis on a phylum **(A)**, class **(B)**, and order **(C)** level.

**Figure 4:**
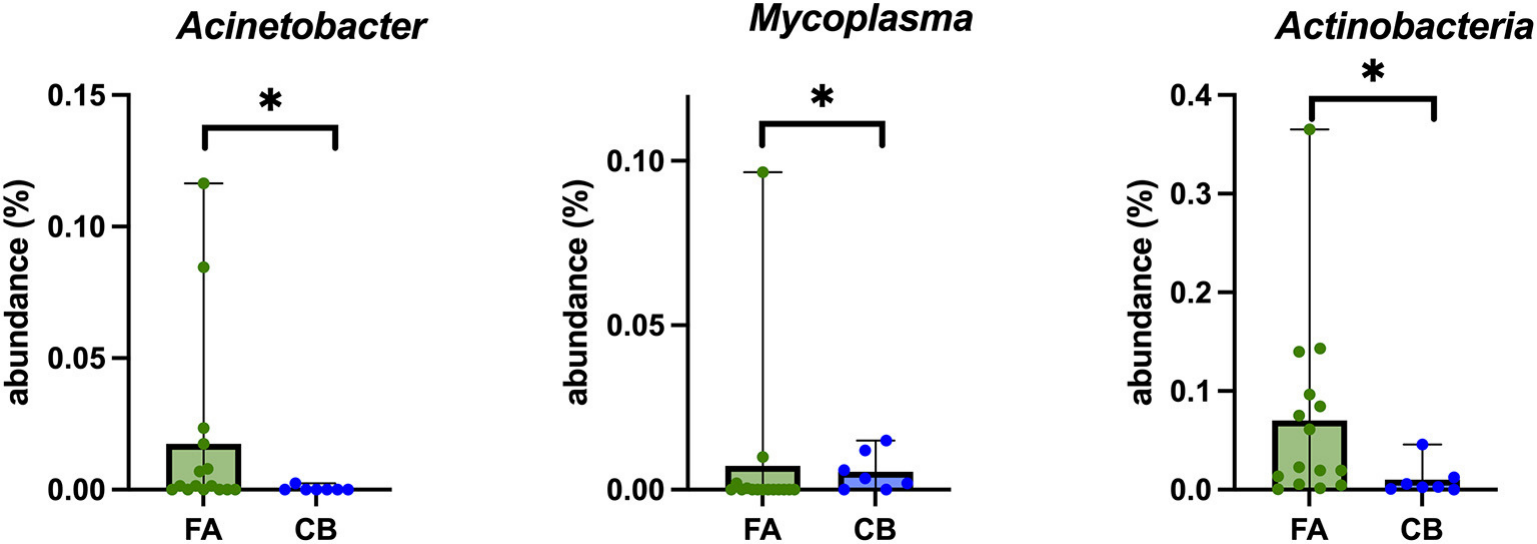
Box plots of bacterial taxonomic groups that were significantly different in BALF-samples of cats with feline asthma (FA; green dots) and chronic bronchitis (CB; blue dots). Median is represented by the upper limit of the colored boxes, range is represented by the upper and lower thin lines. *p < 0.05.

Figure 3 shows the relative composition of the microbiota of all patients on the phylum, class, and order level. At the phylum level, *Bacteroidetes, Proteobacteria, Firmicutes*, and *Actinobacteria* dominated in all cats. At the phylum level, significant differences emerged only for the phylum *Actinobacteria* between the cats with FA and CB. Generally, strong interindividual differences were seen. With 2.29%, the median percentage of *Actinobacteria* was significantly higher in the FA (range 0.05–36.52%) compared to the CB group with 0.3% (range 0–4.58%) (*p* = 0.026) (Figure 4).

At the class level, the groups *Saprospirae, Gammaproteobacteria, Alphaproteobacteria*, and *Epsilonproteobacteria* were found to be the dominant groups. There were no significant differences between the FA and CB group regarding the dominant class groups. At the taxonomic level of order, the dominant orders were *Saprospirales, Pasteurellales, Campylobacteriales*, and *Pseudomonadales*. There was no significant difference between the two groups regarding the dominant orders. At the family level, the most abundant families in both cat populations were *Chitinophagaceae, Pasteurellaceae, Helicobacteriaceae*, and *Xanthomonadaceae*. There were also no significant differences between both groups. Regarding the taxonomic level of genus, the groups belonging to unclassified *Chitinophagaceae*, unclassified *Pasteurellaceae*, unclassified *Helicobacteriaceae*, and the genus *Stenotrophomonas* were frequently represented. Relative abundances of the four most prevalent taxa in the phylum, class, order, family, and genus level can be found in Table 3.

**Table 3:** Relative abundance (expressed in percentage) of the four most prevalent taxa at the levels phylum, class, order, family, and genus in cats with chronic bronchial disease.

	**FA**		**CB**		***p*-value**
**Taxa**	**Median**	**Range**	**Median**	**Range**	
**Phylum**					
*Actinobacteria*	2.29	0.05–36.52	0.30	0–4.58	0.026
*Bacteroidetes*	9.95	0–98.81	85.67	1.99–96.97	0.259
*Firmicutes*	2.49	0–11.59	1.04	0.10–33.73	0.833
*Proteobacteria*	39.35	0.90–94.38	8.76	0.90–84.43	0.324
**Class**					
*Alphaproteobacteria*	3.78	0–43.78	0.15	0–5.42	0.128
*Epsilonproteobacteria*	0.25	0–89.10	0.60	0.25–1.09	0.303
*Gammaproteobacteria*	11.89	0.30–49.7	6.47	0.50–83.08	0.916
*Saprospirae*	2.39	0–98.51	83.98	1.49–96.52	0.245
**Order**					
*Campylobacteriales*	0.25	0–89.10	0.60	0.25–1.09	0.303
*Pasteurellales*	1.19	0–28.31	1.59	0–82.44	0.972
*Pseudomonadales*	1.54	0–19.15	0.55	0.10–41.04	0.549
*Saprospirales*	2.39	0–98.51	83.98	1.49–96.52	0.245
**Family**					
*Chitinophagaceae*	2.39	0–98.51	83.98	1.49–96.52	0.245
*Helicobacteriaceae*	0.25	0–89.1	0.35	0.20–0.70	0.434
*Pasteurellaceae*	1.19	0–28.31	1.59	0–82.44	0.972
*Xanthomonadaceae*	1.49	0–22.14	0.15	0–17.31	0.242
**Genus**					
Unclassified *Chitinophagaceae*	2.39	0–98.51	83.98	1.49–96.52	0.245
Unclassified *Helicobacteriaceae*	0.25	0–89.1	0.35	0.20–0.70	0.434
Unclassified *Pasteurellaceae*	1.19	0–27.71	1.59	0–82.44	0.972
*Stenotrophomonas*	1.04	0–22.14	0.05	0–17.31	0.226

FA, cats with feline asthma; CB, cats with chronic bronchitis.

There were significant differences between both groups for the genus *Mycoplasma* (*p* = 0.048), with a significantly higher proportion in the FA group (median 0%; range 0–9.65%) compared to the CB group (median 0.35%; range 0–1.49%) (Figure 4). However, this higher proportion could be explained by the fact that two cats in the FA group carried both a high proportion of *Mycoplasma*. In general, *Mycoplasma* spp. could be detected in 9/22 (40.9%) cats, in 4/15 cats (26.6%) with FA, and in 5/7 (71.4%) cats with CB. In addition, *Acinetobacter* spp. was also found in a significantly higher proportion in the FA group (median 0.15%; range 0–11.64%) compared to the CB group (median 0%; range 0–0.25%; *p* = 0.049) (Figure 4).

### 3.4. Comparison of taxonomic results between different clinical phenotypes

**Figure 5:**
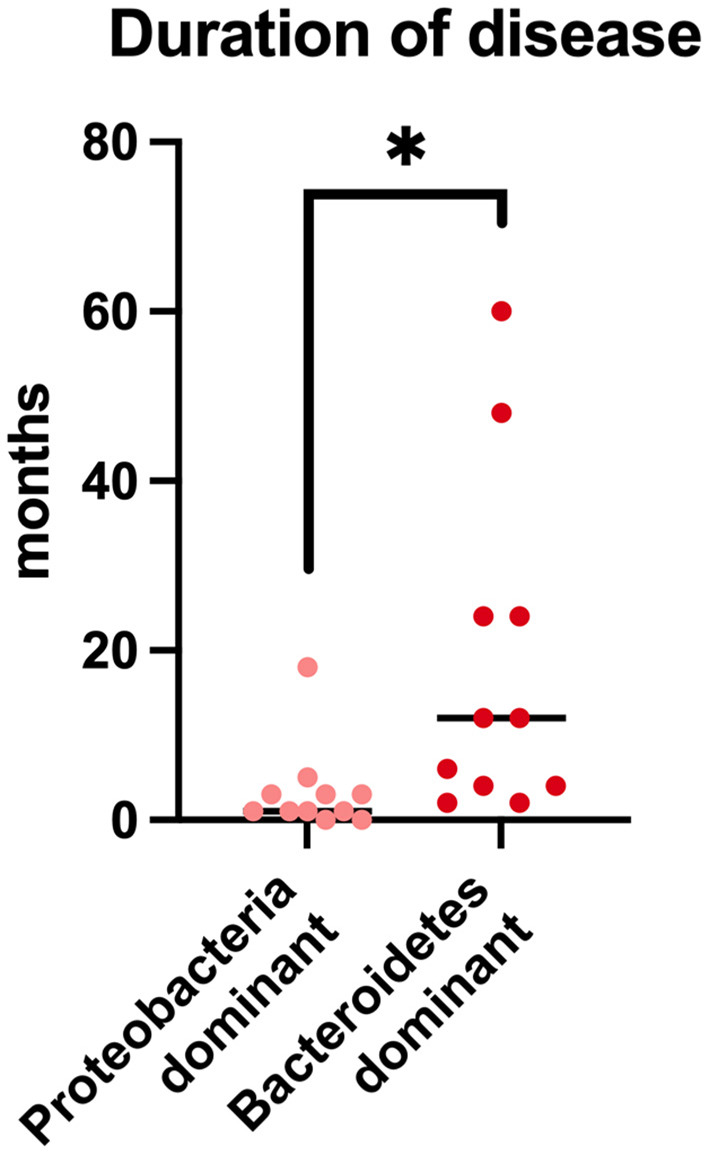
Duration of disease prior to presentation in cats with chronic bronchial disease and predominantly *Protebacteria* in BALF-samples in comparison to cats with predominantly *Bacteriodetes* in BALF-samples. *p < 0.05.

A clear separation was evident between cats that, regardless of the type of pulmonary inflammation, showed a predominance of *Proteobacteria* (11/22; 50%; 9/11 FA, 2/11 CB) or *Bacteriodetes* (11/22; 50%; 6/11 FA, 5/11 CB) as the main component of the microbiota composition at the phylum level. Therefore, data of the cats of these subgroups were investigated in terms of age, body weight, duration of clinical signs before presentation, and clinical signs. It was found that animals dominated by *Bacteriodetes* had significantly longer duration of clinical signs before presentation (median 12 months, range 2–58 months) compared to animals dominated by Proteobacteria (median 1 month, range 1 day to 18 months; *p* = 0.003) (Figure 5). In addition, the animals with a *Bacteriodetes* predominance showed cough as clinical sign significantly more often (11/11 cats) compared to cats with predominance of *Proteobacteria* (6/11 cats; *p* = 0.035). Regarding the other clinical signs and phenotypic data, there were no significant differences between the groups (data not shown).

## 4. Discussion

The present study is the first to compare the composition of the lung microbiome in cats with FA and CB. No significant differences could be detected in the diversity, richness, and evenness of the lung microbiota between the two disease groups. However, there were differences in the composition of individual bacterial groups that are described here for the first time.

The client-owned cat population included in this study appeared to be relatively young, with a median age of 3 years, and included more pedigreed than domestic shorthair cats. Animals in the FA group were slightly younger than animals in the CB group, although this was not significant. This distribution pattern is consistent with findings in other studies in which cats with FA were also slightly younger (19–21). In one study, healthy young cats were shown to have a stronger respiratory response to triggering agents compared with older animals, which may explain the occurrence of FA at a young age (19). Regarding the duration of the disease, there was no difference between the two groups. Besides that, the distribution of clinical signs was also similar between cats with FA and CB. This finding is in line with other publications and underlines that FA and CB are disorders that cannot be distinguished based on clinical signs (25). Moreover, hypotheses have been discussed, that both disorders might be two presentations of one disease complex (46).

In humans, alterations of the microbiota in various respiratory diseases, such as asthma, have been investigated, but results are still limited (13–15, 17, 47–49). In veterinary medicine, few studies have been focusing on the respiratory microbiome (31, 38, 50). Cats with FA are unique in the animal world, because they are the only known species to suffer from eosinophilic obstructive airway disease analogous to humans (51). The composition of the lung microbiota is thought to be based on the immigration of bacteria mainly by subclinical microaspiration from the airways, although other sources, including inhaled air and aspiration of gastric contents, may also make a small contribution. The clearance of members of the lung microbiota then depends, in turn, on the presence and effectiveness of many host-specific factors, including cough reflex, mucociliary clearance, and innate and adaptive immunity (52).

In the present study population, *Bacteroidetes* and *Proteobacteria* were the most abundant components at the phylum level. There was no difference in the proportional distribution of these groups between animals of the FA and the CB group. In humans, it is well-known that a healthy lung microbiome consists mainly of *Bacteroidetes*, and that a shift has been observed in human asthma, leading to a dominance of *Proteobacteria* in this disease in contrast to healthy individuals (47).

In two recently published veterinary studies, cats with experimentally induced and spontaneous FA were studied, and the lung microbiome was evaluated by 16S rRNA sequencing (31, 38). Cats with FA induced by Bermuda grass allergen were evaluated 6 and 36 weeks after induction. The lung microbiome was found to be significantly decreased in richness and beta diversity compared to findings before induction of the allergic response, which was more pronounced after 36 weeks. Thus, this study demonstrates that the lung microbiome appears to change progressively in cats with FA over time. Taxonomically, the number of cats carrying predominantly *Proteobacteria* or *Bacteriodetes* changed as well with an increase of *Bacteriodetes* and a decrease of *Proteobacteria* with increasing chronicity of the disease. In our study, regardless of the type of pulmonary inflammation, it was clearly evident that cats with a predominance of *Bacteriodetes* had been symptomatic for significantly longer periods of time compared to cats in which *Proteobacteria* were dominating. Thus, a change in the lung microbiota in a chronic disease process can be quite well-supported by our data. In addition, all cats with a dominant *Bacteriodetes* colonization showed cough, whereas cats in the group with a dominance of *Proteobacteria* frequently (45% of cases) showed no cough, but wheezing, acute dyspnea and other clinical signs. This finding could be an indication of the chronicity and severity of the disease in cats with an altered microbiome. In another recently published study, the lung microbiome of cats with spontaneous FA was evaluated in comparison to a healthy control group. This investigation also revealed a significant change in the microbiome of diseased compared to healthy cats. While healthy cats were dominated by the phylum *Proteobacteria, Bacteroidetes* predominated in asthmatic patients. Results of this study in accordance with data generated in the present study indicate that an evolution of the microbiota with a shift from the phylum *Proteobacteria* to *Bacteriodetes* can be interpreted as an abnormal change of the microbiome associated with progression of disease. In addition, the healthy control population of the cited study showed a microbiome dominated by Proteobacteria in all included healthy cats which was different from the more diverse composition of the microbiome of the present study in which partly *Proteobacteria* and partly *Bacteriodetes* dominated. In addition, the phylum Proteobacteria in the cited healthy control group appeared to consist mainly of *Pseudomonaceae*. This is quite contrary to the composition of this phylum in the present study where the composition is more diverse and not dominantly composed of *Pseudomonaceae*. From various studies investigating human and animal microbiota, it is known that changes in the microbiota can be a continuous process with a timely progression. Further studies would be desirable to evaluate whether early therapeutic intervention can help to restore normal lung microbiota. In addition, it would be interesting to evaluate the clinical response of cats with respiratory disease, depending on the lung microbiota composition. At present, it remains unclear, if changes of the airway microbiome might have an impact on clinical response of cats.

It was an interesting finding that *Mycoplasma* spp. was found in 41% of cats with chronic bronchial disease by 16S rRNA gene sequencing, whereas all cats had previously been tested negative by a targeted PCR method. In contrast, in culture, PCR, or microbiota analysis in healthy cats *Mycoplasma* spp. could not been detected in the lower airways (43, 47). Human infections with *Mycoplasma* have been shown to contribute to the development of chronic airway signs, exacerbate clinical signs, and complicate the management of asthmatic patients (53). Successful treatment of the infection has improved pulmonary function in asthmatic patients and resulted in an improved quality of life. It has been estimated that the prevalence of *Mycoplasma* in cats suffering from FA and CB can be up to 35% with culture dependent methods (39). Using sequencing methods, *Mycoplasma* spp. could be detected in 35% of cats with naturally occurring FA (38). Since *Mycoplasma* spp. is not considered a commensal symbiont of the lower airways, it must be assumed that in cats with inflammatory bronchial disease *Mycoplasma* spp. either have the ability to invade the lower airways by spreading from the upper airways in order to take advantage of an altered microbiota, or it may have been introduced into the lower airways by extension from the upper respiratory tract (32). Testing for *Mycoplasma* spp. in cats with asthma or CB is recommended due to the links between *Mycoplasma* spp. detection and asthma in humans and the high prevalence of *Mycoplasma* spp. in this and other studies. So far it is unknown, if targeted antimicrobial therapy against *Mycoplasma* spp. or other bacterial species might be beneficial in improving clinical outcomes in cats with chronic bronchial disease.

The study has several limitations. The most significant limitation is the fact that no healthy control group was included for comparison for ethical reasons. Evaluation and comparison of the results were only performed between the two diseased cat populations and compared to results derived from healthy cats described in other publications. Nevertheless, the authors are aware of the fact that that a comparison of results to data generated with a different sequencing set also has limitations.

Only cats without any antimicrobial treatment in the last 4 weeks prior to presentation were included. However, some cats had been treated with antibiotics in the months before. As known from studies in other organ systems, antimicrobial treatment can lead to a significant change in the microbiota of the individual, even when treatment had been given longer than 4 weeks ago. Accordingly, it cannot be excluded that a certain individual microbial composition could have been caused by pretreatments. Furthermore, the group size was small, especially in the CB group. Larger patient populations would be desirable in the future to reevaluate the findings.

## 5. Conclusion

Cat populations with spontaneous FA have the same lung microbiota composition as those with spontaneous CB. Evidence exists that the microbial composition changes with the duration of the disease. Compared to the classical PCR-based detection methods, sequencng methods have been found to be more effective in identifying *Mycoplasma* spp. as a microbial compotent.

## Data availability statement

The datasets presented in this study can be found in online repositories. The names of the repository/repositories and accession number(s) can be found below: https://www.ncbi.nlm.nih.gov/, PRJNA916730.

## Ethics statement

The animal study was reviewed and approved by the Ethics Committee of the Faculty of Veterinary Medicine, Ludwig-Maximilian-University (Number: 119-09-04-2018). Written informed consent was obtained from the owners for the participation of their animals in this study.

## Author contributions

MW contributed to study design, study analysis and statistical analysis, and wrote the manuscript. JW, LH-S, and KW contributed to the study design, sample collection, and revision of the manuscript. JP contributed to study design, cytology of BALF-samples, and revision of the manuscript. JSh, JSu, and RP contributed to sample analysis, statistical analysis, and revision of the manuscript. BS contributed to study design, sample collection, study analysis, and revision of the manuscript. All authors contributed to the article and approved the submitted version.
